# Survival from XDR-TB Is Associated with Modifiable Clinical Characteristics in Rural South Africa

**DOI:** 10.1371/journal.pone.0031786

**Published:** 2012-03-06

**Authors:** Sheela V. Shenoi, Ralph P. Brooks, Russell Barbour, Frederick L. Altice, Daniel Zelterman, Anthony P. Moll, Iqbal Master, Theo L. van der Merwe, Gerald H. Friedland

**Affiliations:** 1 Department of Medicine, Section of Infectious Diseases, AIDS Program, Yale University, School of Medicine New Haven, Connecticut, United States of America; 2 Philanjalo Care Centre, Tugela Ferry, South Africa; 3 Center for Interdisciplinary Research on AIDS, Yale University, New Haven, Connecticut, United States of America; 4 Division of Biostatistics, Yale University School of Public Health, New Haven, Connecticut, United States of America; 5 Church of Scotland Hospital, Tugela Ferry, South Africa; 6 King George V Hospital, Durban, South Africa; Institute of Infectious Diseases and Molecular Medicine, South Africa

## Abstract

**Background:**

Drug-resistant tuberculosis (TB) is a major threat to global public health. Patients with extensively drug-resistant TB (XDR-TB), particularly those with HIV-coinfection, experience high and accelerated mortality with limited available interventions. To determine modifiable factors associated with survival, we evaluated XDR-TB patients from a community-based hospital in rural South Africa where a large number of XDR-TB cases were first detected.

**Methodology/Principal Findings:**

A retrospective case control study was conducted of XDR-TB patients diagnosed from 2005–2008. Survivors, those alive at 180 days from diagnostic sputum collection date, were compared with controls who died within 180 days. Clinical, laboratory and microbiological correlates of survival were assessed in 69 survivors (median survival 565 days [IQR 384–774] and 73 non-survivors (median survival 34 days [IQR 18–90]). Among 129 HIV+ patients, multivariate analyses of modifiable factors demonstrated that negative AFB smear (AOR 8.4, CI 1.84–38.21), a lower laboratory index of routine laboratory findings (AOR 0.48, CI 0.22–1.02), CD4>200 cells/mm^3^ (AOR 11.53, 1.1–119.32), and receipt of antiretroviral therapy (AOR 20.9, CI 1.16–376.83) were independently associated with survival from XDR-TB.

**Conclusions/Significance:**

Survival from XDR-TB with HIV-coinfection is associated with less advanced stages of both diseases at time of diagnosis, absence of laboratory markers indicative of multiorgan dysfunction, and provision of antiretroviral therapy. Survival can be increased by addressing these modifiable risk factors through policy changes and improved clinical management. Health planners and clinicians should develop programmes focusing on earlier case finding and integration of HIV and drug-resistant TB diagnostic, therapeutic, and preventive activities.

## Introduction

Drug-resistant tuberculosis (TB) has emerged as a major threat to global public health. In 2006, a large number of cases of extensively drug-resistant tuberculosis (XDR-TB), newly defined as resistance to isoniazid, rifampin, any fluoroquinolone and an injectable antimicrobial agent (kanamycin, amikacin, or capreomycin), were reported from a district hospital in Tugela Ferry, a rural site in KwaZulu Natal province, South Africa [1]. Initially, 53 XDR-TB cases were characterized by HIV-coinfection with accelerated and near-complete (98%) mortality; median survival from sputum collection was 16 days [1]. XDR-TB has since been reported throughout South Africa [2], in neighboring sub-Saharan African countries and in 58 countries worldwide [3] and jeopardizes both TB and antiretroviral therapy (ART) treatment programmes globally [4]. South Africa is among the countries with the greatest burden of drug-resistant TB including XDR-TB [5]. By year-end 2008, 463 patients from Tugela Ferry had been diagnosed with XDR-TB. Though the mortality rate has fallen, it remains extremely high (82%) and rapid for most patients with a median time to death after sputum collection of 28.5 days [6].

Information regarding survival and outcomes of patients with XDR-TB remains both limited and inconsistent. Accelerated and high mortality from XDR-TB in Tugela Ferry, where HIV-coinfection exceeds 90%, contrasts with reports from other geographic areas where XDR-TB-related mortality in patients without HIV-coinfection, has ranged from 7%–48%[1], [7], [8], [9], [10]. In a more recent series of XDR-TB patients from a TB specialty referral hospital in South Africa, the HIV-coinfection rate was substantially lower (47%) than that reported from Tugela Ferry and 3-month mortality rates were more favorable [11].

Despite considerable attention from public health planners, programme officials, and clinicians regarding the management of XDR-TB, there are limited empirical data regarding the management of XDR-TB and HIV-coinfection and little insight into factors associated with survival. The increase in XDR-TB cases and recently observed improved survival in Tugela Ferry now provides a unique opportunity to determine correlates of survival among XDR-TB patients from a single non-referral community-based hospital. In this study, we focus on modifiable factors that may be associated with improved XDR-TB survival.

## Methods

We performed a retrospective case control study of patients with XDR-TB from Tugela Ferry, South Africa from 2005–2008.

### Setting

Tugela Ferry is a rural, resource-limited community in KwaZulu Natal province, South Africa, with 180,000 traditional Zulu people living in widely dispersed compounds extending over 2000 square kilometers. Most people lack access to potable water (69%) or electricity (69%) and unemployment is high (85%) [12]. The 350-bed district government Church of Scotland Hospital (COSH) and its 15 primary health care clinics provide health care for this district. TB DOTS and HIV services began in 1995 and 2003, respectively. TB incidence is 1054/100,000 [13] and HIV prevalence among pregnant women exceeds 30%[14].

### Ethics Statement

The study was approved by institutional review boards at both University of KwaZulu Natal in Durban, South Africa and Yale University in New Haven, Connecticut. The data reviewed and presented in this study was originally collected in medical records as part of routine clinical care. Informed consent was not requested of patients since recording of data into the medical charts was for the purpose of regular medical care. The ethics committees waived the requirement for informed consent for this retrospective study given that the use of previously collected routine clinical data did not present further risk to patients.

### Management of TB and HIV

Initial TB diagnosis and treatment initiation was based on sputum acid-fast bacilli (AFB) smear, chest radiograph and clinical judgment. Sputum culture was usually obtained for treatment relapse, treatment failure, or persistently positive AFB at the end of the intensive phase of treatment, rather than at primary diagnosis [15]. After recognition of drug-resistant TB in 2006, cultures at COSH could be ordered during the initial TB diagnosis. Category 1 TB treatment using isoniazid (INH), rifampin (Rif), ethambutol, and pyrazinamide for the 2-month intensive phase was followed by a 4-month continuation phase using INH/Rif [16]. Ziehl-Neelsen microscopy of sputum was performed locally at COSH while auramine smear, culture, and drug susceptibility testing (DST) (isoniazid, rifampin, ethambutol, streptomycin, ciprofloxacin, kanamycin) were performed at the provincial referral laboratory in Durban using previously described methods [1]. Typically, culture and DST results were available after 2–3 months.

Microbiologically-confirmed XDR-TB patients were traced by the local DOTS office and if alive, referred to the provincial TB specialty referral hospital, King George V (KGV), in Durban, to initiate XDR-TB treatment - a process requiring a minimum of several months from diagnostic sputum collection. The standardized intensive phase regimen for XDR-TB consisted of ethambutol, pyrazinamide, ethionamide, cycloserine or terizidone, para-aminosalicylic acid, and capreomycin - supervised within an inpatient unit for a minimum of 6 months. The latter two agents became available only in late 2007. Capreomycin injections were discontinued during the continuation phase, and the remaining oral regimen maintained for a minimum of 18 months with monthly assessments in Durban. South African guidelines through 2009 provided ART for individuals whose CD4 cell count was less than 200 cells/mm^3^, using regimens combining stavudine and lamivudine with either efavirenz or nevirapine [15]. An alternative regimen of once-daily lamivudine, didanosine and efavirenz was used in selected cases [17]. HIV and TB care were integrated within the same clinic in Tugela Ferry while physically and administratively distinct within KGV.

### Study Subjects and Definitions

All patients meeting standardized microbiological definition of XDR-TB identified from culture records and TB DOTS offices for the 4-year period from January 1, 2005 through December 31, 2008 were eligible for inclusion. Survival was defined as living >180 days from the date of sputum collection confirming XDR-TB. Non-survivors were those XDR-TB patients living <180 days from sputum collection. The two groups were compared to identify modifiable factors associated with survival at 180 days. The median survival was calculated for both groups based on known outcome data censored on March 31, 2009.

**Figure 1:**
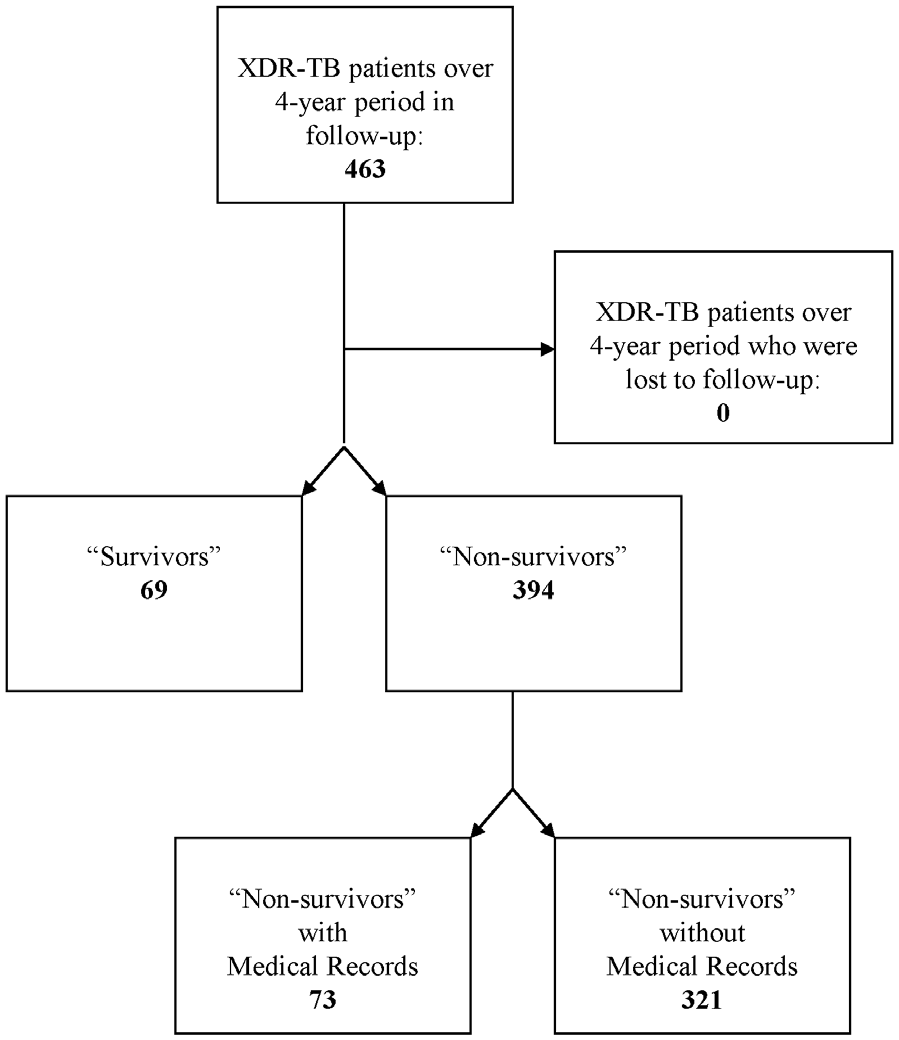
Selection of Charts for Review.

Of the 463 eligible patients with XDR-TB, 69 (14.9%) met the survival case definition. From the remaining 395 non-survivors, subjects were reviewed based on availability of medical records. Seventy-three non-survivors' charts were available for review, resulting in 142 patients with XDR-TB available for analysis (Figure 1).

Inpatient and outpatient records were reviewed using a standardized data collection instrument that included demographic, clinical features, laboratory findings, and health-related outcomes. CD4 counts were considered as baseline if collected within 90 days of initial sputum confirming XDR-TB. TB cases were defined as “initial” if they had not previously been treated for TB and “recurrent” if they had previously completed a course of treatment before their XDR-TB diagnostic sputum collection. Cases that had prior confirmed MDR-TB and were receiving MDR-TB therapy were included. An additive laboratory clinical severity index (LCSI) was created from data collected at the time of sputum collection, consisting of abnormal values: hemoglobin (<13 gm/dL); creatinine (>53 mg/mL); albumin (<35 g/mL); and alkaline phosphatase (98 IU/mL) to explore the relationship of these indicators individually and collectively from sputum collection to the 180-day survival point. A value of “1” was assigned for each result that was outside the limits of normal and added together resulting in a count of possible observed abnormalities from 0 to 4.

### Analytic Plan

**Figure 2:**
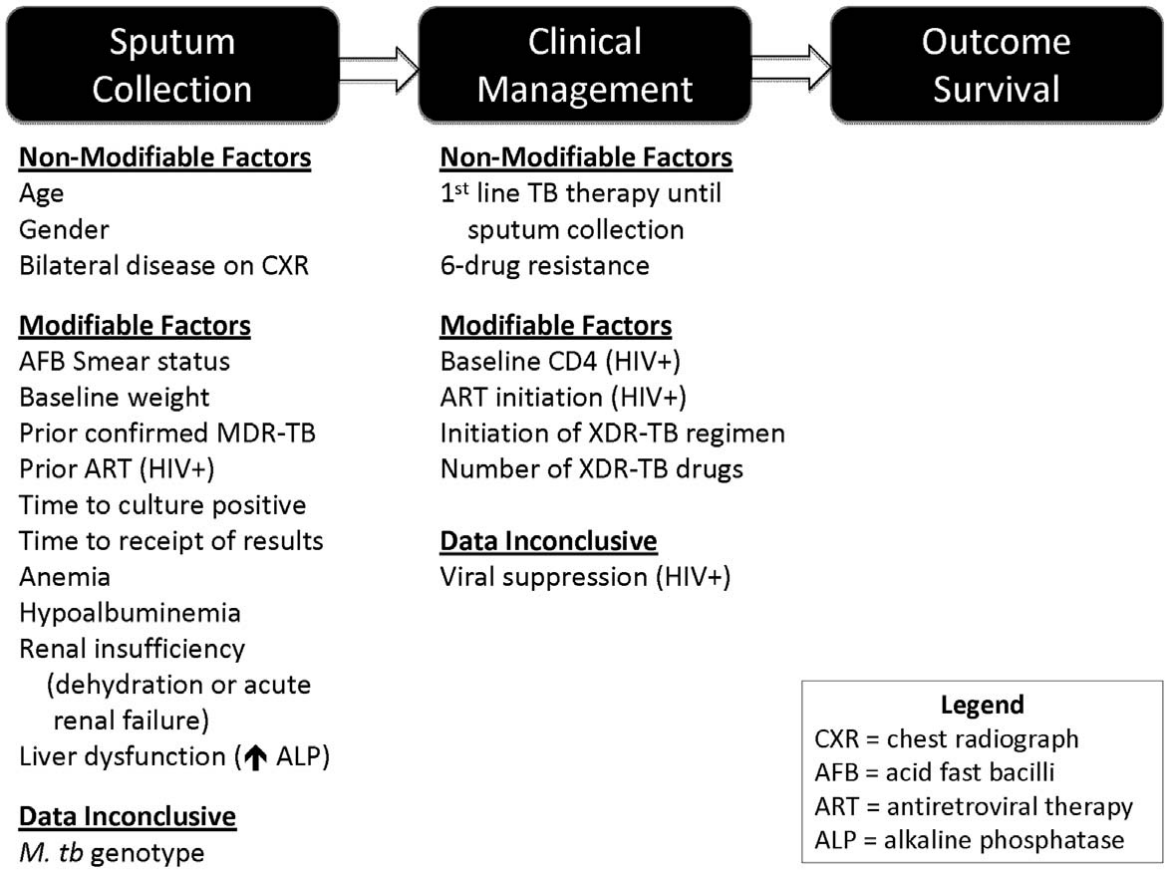
Modifiable & Non-Modifiable Clinical Characteristics of XDR-TB Patients.

Frequencies for demographic characteristics and medians with interquartile ranges (IQR) for clinical and laboratory parameters were calculated. The primary outcome was survival from XDR-TB. Student's t-test, Kruskal-Wallis, and Mantel-Haenszel Chi square of potential independent variables were assessed using SAS version 9.1 (Cary, NC) to compare survivors and non-survivors. The mean LCSI score was compared for survivors and non-survivors. Given several variables with a large amount of missing data, the missing completely at random (MCAR) assumption was assessed for the variables in the final multivariate model using Little's test (SPSS 18.0). When >5% of values were missing, they were suggested to be missing completely at random and were not related to survival (p>0.71). [17] Based on the interest in identifying modifiable factors affecting survival, we first characterized all variables affecting XDR-TB survival as modifiable or non-modifiable (Figure 2); only those modifiable variables significant at the p<0.1 on univariate analysis were entered into the multivariate logistic regression analysis. XDR-TB treatment variables were determined to be confounders with the definition of survival (alive at 180 days) and were not included in multivariate analysis. A backward stepwise elimination multivariate logistic regression was applied using the ‘step’ function for model selection in R using the Generalized Linear Model framework [18] (version 2.7, Foundation for Statistical Computing, Vienna, Austria, http://www.r-project.org). The standard logit link function and the Akaike Information Criteria (AIC) in *R* were utilized for model selection; the model with lowest AIC was selected. [19] The Hosmer-Lemeshow and the Nagelkerke R^2^ tests were used to assess goodness of fit of the final model. Four modifiable variables, found to be significantly related to 180-day survival among HIV-infected patients, were subjected to Kaplan-Meier estimates for the dependent variable of survival, using the “survfit” with the *R* “survival” package. [20] A logrank test in *R* was used to test the difference; proportional hazard calculation was performed to illustrate the increasing hazard of death in XDR-TB patients with increasing LCSI score.

## Results

Demographic characteristics of the 142 selected subjects did not differ from the 463 total XDR-TB patients diagnosed during this period (data not shown). Non-survivors (n = 73) reviewed were not significantly different in age or gender from overall non-survivors (n = 394). The median survival after data censoring was 565 days (IQR 384–784) in survivors and 34 days (IQR 18–90) in non-survivors (p<0.0001). Sixty-two (43.7%) XDR-TB patients presented with a first episode of TB, while 79 (55.6%) previously had completed therapy for drug-susceptible TB. Of the non-survivors reviewed, 42 (57.5%) had already died by the time the culture and DST results were received. Of survivors at the point of censoring, 52 (75%) had already reached 1 year of observation and 20 (28.9%) had been observed for 2 years of follow-up.

There was no significant difference in proportion of women, (64%), median age (34 years, IQR 30–35), or proportion HIV-infected (90.8%) between survivors (n = 61, 89.7%) and non-survivors (n = 68, 94.4%). Survivors and non-survivors did, however, differ on a number of important characteristics (Table 1). Non-survivors were significantly more likely than survivors to present with XDR-TB as their initial TB episode (53.4% vs 33.3%, p = 0.02). Laboratory parameters differed significantly between survivors and non-survivors. Survivors demonstrated significantly better laboratory values and LCSI scores, including higher hemoglobin and albumin levels, fewer renal and liver abnormalities and fewer metabolic disturbances. The mean LCSI was 1.28 in survivors and 2.22 in non-survivors (p<0.0001). In addition, survivors were more likely to have negative auramine smears when compared to non-survivors, and survivors' sputum cultures grew more slowly with results reaching clinicians later than those of non-survivors. Survivors had significantly less six-drug resistance on standard drug susceptibility testing.

**Table 1:** Demographic, Clinical, Sputum, and TB& HIV Treatment Characteristics Among Survivors & Non-survivors of XDR-TB (n = 142).

	Survivors n = 69(% or IQR)	Non-survivors n = 73 (% or IQR)	p-value
*Demographic Characteristics at Diagnostic Sputum Collection*			
Median age (years)	34 (30–45)	34 (30–40)	0.83$
Women	40 (57.9)	50 (68.5)	0.19§
*Clinical & Laboratory Characteristics at Diagnostic Sputum Collection*			
HIV-infected (n = 140)	61 (89.7)	68 (94.4)	0.30§
Median days of survival	565 (384–774)	34 (18–90)	<0.0001€
Median months of TB symptoms prior to TB diagnosis (n = 102)	3.0 (1.5–6)	3.0 (1–5)	0.24€
First episode of TB	23 (33.3)	39 (53.4)	0.02§
Median weight (kg) (n = 123)	51 (45.6–58.5)	47.5 (41–55)	0.11€
Bilateral disease on chest x-ray (n = 104)	34 (70.8)	30 (53.6)	0.07§
• Median hemoglobin (g/dL) (n = 109)* Proportion of patients with abnormal hemoglobin**	10.3 (9–11.9) 37/41 (90.2)	8.8 (7.4–10.3) 68/68 (100)	<0.001€
• Median creatinine (umol/L) (n = 101)* Proportion of patients with abnormal creatinine	67.0 (54.1–89) 14/38 (36.8)	80.0 (65.8–116) 31/63 (49.2)	0.008€
• Median alkaline phosphatase (IU/mL) (n = 60)* Proportion of patients with abnormal alkaline phosphatase	104.5 (78.9–143) 16/21 (76.2)	148 (86–212.6) 25/39 (64.1)	0.08€
• Median albumin (g/dL) (n = 62)* Proportion of patients with abnormal albumin	29.3 (25–33) 21/23 (91.3)	23.45 (18.1–28.3) 38/39 (97.4)	0.001€
Mean Laboratory Clinical Severity Index (LCSI)	1.28	2.22	<0.0001$
*Sputum Characteristics at Diagnostic Sputum Collection*			
Negative auramine smear (n = 139)	42 (61.8)	15 (21.1)	<0.0001§
Median weeks to positive sputum culture (n = 137)	3 (2–3)	2 (2–2)	0.0002€
Median days to receipt of culture with DST results (n = 106)	71 (61–92)	61 (51–75)	0.004€
Resistance to all 6 drugs tested (n = 142)	49 (71)	62 (84.9)	0.04§
*TB Therapy*			
Receiving TB therapy at time of diagnostic sputum collection	42 (60.8)	53 (72.6)	0.21§
Prior confirmed MDR-TB	9 (13)	3 (4.1)	0.056§
Mean number drugs in XDR-TB regimen (n = 65)	6.25	5.6	0.22$
*HIV Characteristics & Therapy*			
Baseline CD4 at diagnostic sputum collection (cells/mm^3^) n = 56	181 (60–260)	83.5 (32–169)	0.02$
Ever initiated ART	49 (80.3)	28 (41.2)	<0.0001§
On ART prior to diagnostic sputum collection	30 (61.2)	20 (71.4)	0.37§
Median months on ART prior to diagnostic sputum collection	4.2 (2.0–10.2)	3.4 (1.2–6.8)	0.22€

* not individually included in regression analysis.

** p<0.05.

$ ttest.

§ Chi-square.

€ Kruskal-Wallis.

XDR-TB patients received first-line TB therapy for a median duration of 16.9 weeks prior to sputum collection with no difference between survivors and non-survivors. Among the 95 patients who were already receiving first-line therapy at the time of sputum collection, only 26 (27%) received streptomycin prior to sputum collection though the majority of reviewed XDR-TB patients (n = 120, 84.5%) demonstrated streptomycin resistance on DST. Only 12 (8.5%) of the 142 XDR-TB patients received treatment for culture-confirmed MDR-TB prior to the XDR-TB sputum collection. With regards to XDR-TB therapy, nearly all survivors (95.7%) lived long enough to be referred to the provincial TB referral hospital (KGV) for standardized XDR-TB therapy. In contrast, 72.6% of non-survivors died before they could be referred and receive specific therapy. Among the study cohort, 86 (60.5%) patients initiated XDR-TB therapy a median of 2.9 months (2.3–3.8) after sputum collection; documented conversion to negative sputum culture occurred in 32 (47%) survivors and in only one of the non-survivors.

**Figure 3:**
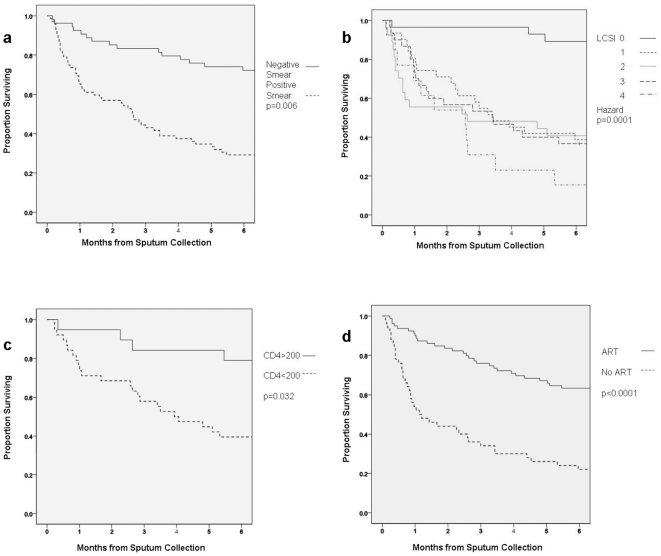
Kaplan-Meier Estimates of XDR-TB Survival in HIV-Infected Patients (Months), n = 129. a. Probability of survival according to auramine smear status from time of sputum collection b. Probability of survival according to severity of organ dysfunction at sputum collection c. Probability of survival according to baseline CD4 count at sputum collection d. Probability of survival according to use of ART from the time of sputum collection.

HIV-specific measures and ART treatment experience differed between survivors and non-survivors. Median baseline CD4 cell counts of survivors were significantly higher than that of non-survivors (181 vs 83.5 cells/mm^3^, p = 0.02). Among XDR-TB patients with HIV-coinfection, survivors were almost twice as likely to ever receive ART compared to non-survivors (80.3% vs 41.2%, p = 0.02). In Kaplan-Meier analyses, CD4 count >200 cells/mm^3^ and ART initiation were significantly associated with survival (Figure 3). Among the 31 survivors who initiated ART and had repeat CD4 cell count testing after XDR-TB diagnosis, the median CD4 cell count increased 155 cells/mm^3^ (IQR 78–201). Among the 33 survivors who had subsequent HIV viral load testing, 23 (69.7%) were undetectable (VL<25 copies/mL).

Multivariate regression analysis was performed for all XDR-TB patients regardless of HIV status, demonstrating that negative auramine sputum smear, the number of weeks to a positive sputum culture, and a low LCSI score (less organ dysfunction) were associated with survival. For patients with XDR-TB and HIV coinfection, multivariate regression analysis revealed several independent correlates of survival, including negative auramine sputum smear, CD4>200 cells/mm^3^ at diagnostic sputum collection, and the receipt of ART (Table 2). Though highly significant on univariate analysis, the LCSI, a marker of clinical severity, approached significance on multivariate analysis (p = 0.056). Kaplan-Meier probabilities of survival differed for each modifiable factor (Figure 3).

**Table 2:** Regression Analysis Associated with Survival among XDR-TB patients with HIV-coinfection (n = 129).

Variable	Crude Odds Ratio (CI)	Adjusted Odds Ratio (CI)	Adjusted ORp-value (Wald's)
Negative auraminesmear result	7.78 (2.34, 25.85)	8.37 (1.84, 38.21)	0.006
Abnormal LCSI (Hemoglobin, Creatinine, Alkaline phosphatase, Albumin)	0.48 (0.29, 0.79)	0.48 (0.22, 1.02)	0.056
CD4 count >200 cells/mm^3^ at diagnostic sputum collection	5.5 (1.52, 19.85)	11.53 (1.11, 119.32)	0.04
Received antiretroviral therapy	5.62 (1.35, 23.52)	20.9 (1.16, 376.83)	0.04

## Discussion

To our knowledge, this is the largest sample of XDR-TB patients from a community-based, non-specialty hospital setting in sub-Saharan Africa. Although only 69 of the first 463 XDR-TB patients lived to 180 days (14.8%), this increase in the number of survivors compared to the earliest reports of XDR-TB at this site has allowed for an in-depth examination of correlates of their survival.

We identified an array of modifiable clinical and laboratory characteristics independently associated with 180-day survival. Not surprisingly, less advanced TB disease, reflected by higher rates of negative sputum microscopy, was associated with survival as was less advanced HIV disease, reflected by higher CD4 cell counts. In addition, less organ system dysfunction, manifested by lower clinical severity score, and concomitant ART were associated with survival, the latter being strongly correlated. These modifiable factors, if addressed by earlier TB diagnosis, timely prescription of ART and addressing end-organ damage manifest by abnormal laboratory values, could improve XDR-TB survival even in the absence of new antituberculosis medications.

Notably, the proportion of XDR-TB patients surviving to six months in this study still remains considerably lower than other reports. There are several possible reasons for this. First, unlike other series, including those from other parts of South Africa, [11], [21] this study reviewed survival from the community hospital's perspective, where the XDR-TB diagnosis was made and not from that of the specialty referral center to which patients would have to be transferred for XDR-TB treatment, thereby averting survival or lead time bias. Since these data demonstrate that a substantial proportion of patients die before referral to TB specialty centers and before XDR-TB therapy is even possible, this study gives a more accurate picture of the overall mortality from XDR-TB. The long lag time from suspected TB diagnosis to microbiological confirmation of XDR-TB and institution of appropriate therapy contributes to mortality and underlines the great need for rapid TB diagnostics, particularly at the community level.

Secondly, unlike reports from other geographic regions, this sample is overwhelmingly (91%) HIV-coinfected, with advanced immunosuppression. Reports from Peru [7], Russia [8], Korea [9], [10], and Lithuania [22] describe more favorable survival rates, but in these series there were no or very few HIV-coinfected patients, suggesting the key role of HIV-induced immune suppression on XDR-TB mortality. Intermediate survival rates have been reported in two other studies from South Africa, but HIV prevalence was lower (47% and 70%, respectively) and CD4 counts were substantially higher (273 and 200 cells/mm^3^, respectively) than in the present series. [11], [21] Thus, these data strongly support that not only HIV infection, but the degree of immunosuppression, contributes to XDR-TB mortality, suggesting that earlier identification of HIV could improve outcomes in coinfected patients.

Last, unlike other studies, only a small proportion of patients in this series had been previously treated for confirmed MDR-TB (8.5%). Series with higher rates of previous MDR-TB treatment are likely to have monitored these patients more closely, possibly contributing to longer survival and introducing observation bias into survival estimates. Furthermore, the high proportion of patients for whom XDR-TB was the first diagnosis of TB and the low proportion with prior MDR-TB in this study suggests most XDR-TB infections were acquired through primary transmission. This is supported by the high proportion of streptomycin resistance found among those who had not previously received this medication. This differs markedly from another recent report from South Africa in which most (72%) XDR-TB cases likely resulted from acquired resistance during previous therapy for confirmed MDR-TB [11]. This distinction emphasizes the importance of determining the etiology of XDR-TB in different geographical regions in order to prioritize appropriate management and prevention strategies [23].

### Opportunities for policy, programmatic, and clinical intervention

Assessment of laboratory tests conducted at the time of sputum collection identified several abnormalities for which relatively simple clinical interventions could potentially improve survival. The development of the composite LCSI in this study is exploratory; thus the findings related to its use are provisional but warrant further prospective evaluation. Though clinically intuitive that dysfunction in multiple organ systems conveys a worse prognosis, we confirm this for the first time among XDR-TB patients. Improving nutritional supplementation for hypoalbuminemia, transfusion for clinically significant anemia, and correction of hypovolemia in patients with elevated creatinine, if applied early and aggressively, may contribute to improved survival. In addition to clinical interventions to modify high mortality rates, the advanced stage of both TB and HIV can be modified and survival potentially improved. Advanced TB and HIV disease were associated with XDR-TB mortality within 180 days in this study, underlining the need for earlier case finding in health care and community settings, use of rapid TB diagnostic tests such as GenXpert [24], and earlier therapy for both conditions. Data from this study suggest that there were ample opportunities for earlier XDR-TB diagnosis and treatment; diagnostic sputum culture was not obtained until a median of four months after initiation of first-line TB therapy. Opportunities were also likely missed for both earlier HIV testing and initiation of ART.

Reliance on the traditional TB diagnostic positive AFB smear missed half of the XDR-TB patients in this study and did so in nearly 80% of non-survivors. Known to be less sensitive, particularly in HIV-infected patients, an AFB smear that is negative should not dissuade clinicians from ordering sputum culture and DST in patients clinically suspected of having TB. Particularly where MDR-TB and XDR-TB are prevalent, and until newer molecular tests are widely available, culture should be ordered during the initial assessment [25]. In this series, waiting until patients had failed first-line antituberculosis therapy before ordering a culture had disastrous consequences. The findings support the current South African TB guidelines [15] recommending smear and culture be sent simultaneously in HIV-infected patients suspected of active TB. This recommendation, however, is followed infrequently and operational research is urgently needed to improve implementation while awaiting the availability of new diagnostic methods [24], [26].

From a treatment perspective, the provision of ART either before sputum collection or after diagnosis of XDR-TB was significantly associated with survival. It is important to note that the retrospective study design and the case definition of survival limit the interpretation of the data and the question of whether ART improves survival or whether survival simply leads to more opportunity for ART initiation should be studied prospectively. Nonetheless, this finding validates other retrospective observational studies with less immunosuppressed HIV-infected populations at referral centers [11], [21], suggesting that the effect of ART on survival from XDR-TB is important. Furthermore, cohort studies and randomized controlled trials confirm that ART during treatment of drug-susceptible TB in coinfected patients reduces mortality [27], [28], [29], [30]. Indeed, other factors associated with survival in the current study including that early TB disease manifested by less severe disease and early HIV disease manifested by high CD4 counts, also echo previous studies of drug susceptible TB in South Africa [31], [32], [33], [34]. Thus, ART may be even more important in the setting of XDR-TB where few if any effective antituberculosis therapies are available. In settings where both HIV and TB are endemic and especially where XDR-TB results from primary transmission, widespread and earlier ART initiation may significantly reduce XDR-TB incidence and may contribute to improved survival. The WHO has promoted the “3 I's” for TB control: infection control, isoniazid preventive therapy, and intensive case finding [35]. Accumulating data including those presented here demonstrate that ART is so critical to management of TB/HIV-coinfected patients that “Initiation of ARVs” should be considered the 4^th^ ‘I’.

Clinical management of XDR-TB in KwaZulu Natal province and elsewhere is provided by experts in specialized TB referral centers. Antiretroviral therapy, however, is often managed separately by HIV specialists and is not well integrated with TB care. Convergence of TB and HIV epidemics, however, requires careful coordination to optimize outcomes for both conditions [36], [37]. Concomitant management of drug-resistant TB and HIV have been recognized within international guidelines [38] and the South African 2010 Guidelines [39] now make substantial strides in expanding ART access to all patients with drug-resistant TB. Diagnostics, staging, including CD4 cell count and viral load testing simultaneous with sputum smears and cultures, and therapy for both drug susceptible and drug-resistant TB and HIV-coinfection should ideally occur in the same setting, by the same practitioners. In one such integrated MDR-TB/HIV model of care in South Africa, preliminary results show feasibility, patient acceptability, medication tolerability, and excellent outcomes [40]. Further study is needed but more widespread implementation of this integrated strategy is warranted.

We recognize limitations in our study. First, retrospective chart reviews may introduce bias due to inadequately recorded or missing clinical information. For the variables in the final multivariate model of HIV+ patients, however, data was not disproportionately missing among survivors and nonsurvivors. Second, potential sampling bias may have occurred since only a portion of all non-survivors were reviewed. If oversampling of one or more characteristics occurred as a result, the correlates associated with survival may be misleading. We do know, however, that those reviewed were demographically representative of the entire population of XDR-TB patients. Last, we recognize that case control studies, such as the present one, can only provide associations and cannot conclude causality. Additionally, in this study, the creation of two patient groups, survivors and nonsurvivors, may have artificially contributed to differences observed in the study results. Prospective studies are needed to confirm the actual effect of the modifiable characteristics identified in the present study on survival.

Despite these limitations, this study represents the largest sample of XDR-TB patients from a community-based, non-referral hospital, contributes new information regarding the correlates of survival, and identifies modifiable factors that are amenable to improvement through changes in policy and clinical management of patients with or at risk for XDR-TB. In order to improve survival, health care administrators and TB and HIV clinicians must work collaboratively to identify patients earlier, and integrate diagnostic, therapeutic, and preventive activities.
